# Development of health-related quality of life and subjective health complaints in adults born extremely preterm: a longitudinal cohort study

**DOI:** 10.1186/s12955-022-02018-5

**Published:** 2022-07-23

**Authors:** Merete Røineland Benestad, Jorunn Drageset, Geir Egil Eide, Maria Vollsæter, Thomas Halvorsen, Bente Johanne Vederhus

**Affiliations:** 1 Department of Children and Youth Clinic, Haukeland University Hospital, Bergen, Norway; 2 Department of Global Public Health and Primary Care, University of Bergen, Bergen, Norway; 3 The Faculty of Health and Social Sciences, Western Norway University of Applied Sciences, Bergen, Norway; 4 Department of Clinical Science, Faculty of Medicine, University of Bergen, Bergen, Norway; 5 Centre for Clinical Research, Haukeland University Hospital, Bergen, Norway

**Keywords:** Quality of life, SF-36, Infant, extremely premature, Self-rated health, Subjective health complaints, Preterm adults, Longitudinal cohort

## Abstract

**Purpose:**

To study development trajectories to 34 years of age of health-related quality of life (HRQoL) and subjective health complaints in extremely preterm (EP) born subjects with and without disability, and to compare with term-born controls.

**Methods:**

A Norwegian longitudinal population-based cohort of subjects born in 1982–85 at gestational age ≤ 28 weeks or with birth weight ≤ 1000 g and matched term-born controls completed the Norwegian version of the Short Form Health Survey-36 at ages 24 and 34 and the Health Behaviour in School-aged Children–Symptom Checklist at ages 17, 24 and 34 years. Data were analysed by unadjusted and adjusted mixed effects analyses with time by subject group as interaction term.

**Results:**

A total of 35/49 (73%) surviving EP-born and 36/46 (78%) term-born controls participated at this third follow-up. EP-born subjects with severe disability reported clinical significant lower mean score in all domains compared to the term-born controls. Healthy EP-born subjects reported significantly lower mean scores for vitality, role emotional and mental health, and significantly higher mean score for total and psychological health complaints compared to term-born controls. There were no significant interactions with age regarding HRQoL and somatic health complaints, while there were significant differences in psychological health complaints; the EP-born scored higher at age 24 and lower at age 34.

**Conclusions:**

EP-born adults at age 34 reported inferior HRQoL versus term-born peers, especially in the mental health domains, indicating that the negative differences observed at 24 years remained unchanged.

## Introduction

Since the 1980s survival after extremely preterm (EP) birth has gradually become the rule rather than the exception in high-income countries. Currently, more than 90% survive birth at 27 weeks gestation, which is considered cut-off for being labelled EP, and these infants now constitute 1 in 200 children growing up [1]. Birth at this early stage of pregnancy implies that growth and development normally taking place in a protected intrauterine environment, instead must take place in a neonatal intensive care unit (NICU). Survival often requires comprehensive and invasive intensive care measures which also may be harmful to immature and highly vulnerable infants. Additionally, preterm birth happens for a reason, and the infant carries the burden of whatever pathology that led to the early delivery. Childhood and adolescent consequences of this scenario are fairly well-known, whereas the life-long implications are virtually undescribed, simply because their high survival rates are so recent achievements [2–4].

Several studies have reported cognitive and social limitations, neurosensory deficiencies, mental health problems, psychiatric disorders, and pulmonary, cardiovascular and metabolic abnormalities in EP-born children and adolescents [5–9]. Low gestational age (GA) and low birthweight (BW) have been linked to low educational levels, special educational needs, low income and unemployment [10–16]. These issues all affect health-related quality of life (HRQoL), a concept that refers to the relationships between an individual’s health and ability to function and their perceived well-being [17]. HRQoL is multidimensional and includes domains related to physical, mental, emotional and social functioning as well as the social context in which people live, and is acknowledged by major governmental bodies as a fundamental measure of health [18]. The knowledge on HRQoL in EP-born young adults is slowly increasing, but the data is equivocal and development beyond 30 years of age still uncharted territory [14, 19, 20]. In 2008, a systematic review concluded that the effects of preterm birth on HRQoL seem to diminish over time [19]. Later publications have challenged this notion, reporting inferior HRQoL in preterm born young adults, and a recent review from 2020 concluded it was not possible to conclude on this issue [20–22]. When it comes to EP-born with disabilities, it is reported inferior HRQoL vs. EP-born without disabilities and vs. term-born (TB) controls [23].

We have previously reported on HRQoL and subjective health complaints at 17 and 24 years of age in a population based Norwegian cohort born EP. At age 17, the EP-born did not differ from their term-born peers; however, when faced with the challenges of adult life at age 24, their mental and social HRQoL had deteriorated and psychological health complaints had increased [24]. The present study is an extension of this study, performed at 34 years of age. We aimed to (1) describe self-perceived HRQoL and subjective health complaints at 34 years of age in EP-born subjects with and without disability and term-born controls, and (2) investigate and compare longitudinal development of HRQoL from 24 to 34 years of age, and subjective health complaints at 17, 24 and 34 years.

## Methods

### Study design and participants

This was a longitudinal population-based study. All subjects born *by mothers living within a defined area in western Norway (the counties Hordaland and Sogn og Fjordane) during the period* January 1982 and December 1985 at GA equal to or below 28 weeks or with BW equal to or below 1000 g where invited and included. *Eligible individuals were identified* based on the birth and admission protocols at the NICU of Haukeland University Hospital, the only unit in the region treating EP-born children. The temporally nearest term-born child of the same gender with BW between three and four kilograms (Norwegian 10–90 centiles) were invited as control. If that subject declined, the next born subject was approached, and so on until one term-born child was recruited for each enrolled EP-born.

The first follow-up took place in 2001–2002 at 17 years, the second was conducted during 2008–2009 at 24 years, and the third during 2018–2020 at 34 years of age. All assessments were performed at Haukeland University Hospital, where participants went through advanced studies of lung and exercise capacity [25, 26] and completed the questionnaires. Some few participants (n = 3) completed the questionnaires at home and returned by post.

### Measures

The questionnaires applied at the third follow-up at age 34 years covered the same topics as the previous two follow-ups at 17 and 24 years.

#### Socio-demographic and clinical data

The information on socio-demographic data was obtained from a custom-made questions used in Norwegian population studies (http://www.hunt.ntnu.no). Educational level had originally a five-point response option with college/university more than 4 years as the most advanced. Employment had originally four response options (working, student, unemployed, or disability pension), whereas living arrangement had two response options (single or married/cohabitant). For the purpose of the statistical analyses in these relatively small populations, the categories were dichotomized. The medical history was obtained from the participants themselves and from hospital records.

#### Short-Form 36-Item Health Survey (SF-36)

HRQoL was measured using the Short-Form Health Survey (SF-36) version 1.1 at age 24 and the RAND-36 survey at age 34 [27]. The RAND-36 questionnaire was developed by the RAND Corporation [27]. It is considered equivalent to the SF-36, except minor differences regarding the scoring procedure of the two sub-scales “general health” and “bodily pain”; still, with extremely high correlation between SF-36 and RAND-36 (*r* = 0.99) [28]. For the purpose of this article, RAND-36 is hereafter referred to as SF-36. The questionnaire is a generic measure assessing self-perceived functional health and well-being through eight health domains: physical functioning (10 items), role-physical (four items), role-emotional (three items), bodily pain (two items), general health (five items), vitality (four items), social functioning (two items), mental health (five items) and one item assesses the perceived change in health status. Except for the two role-functioning scales with dichotomized response choices, the responses are rated along a three to six-point Likert-type scale. The preceding four weeks constitute the recall period, except for physical functioning and general health, which pertain to the current status. The raw scores for each SF-36 sub scales were based on the mean of valid items if at least half of the items in each scale were valid, and then linearly transformed into a scale from 0 to 100, with higher scores indicating better functional health and well-being [17, 27]. Generally; a change of 5–10 points on a 0–100 scale is considered clinically significant [29]. The questionnaire is a broadly evaluated health status instrument with good reliability and validity [27]. The translated and validated Norwegian version was applied, which was tested for internal consistency by Cronbach’s alpha and floor and ceiling effect [30, 31].

#### Health behaviour in school-aged children-symptom check list (HBSC-SCL)

Participants’ subjective health complaints were measured using the Health Behaviour in School-aged Children—Symptom Check List (HBSC-SCL), which assesses the occurrence of four somatic (headache, abdominal pain, backache, and feeling dizzy) and four psychological symptoms (feeling low/depressed, irritable/ bad tempered, nervous and sleeping difficulties) [32]. The participants were asked to rate the frequency of symptoms experienced in the past 6 months. Each item was assessed on a 5-point response scale ranging from daily (4) to rarely/never (0). Two sub-scores (0–16) and a total sum-score (0–32) were calculated, higher scores indicate more symptoms [32]. We applied the translated and validated Norwegian version [33], that has revealed satisfactory reliability in test–retest analyses, ranging from 0.70 to 0.80.

### Statistical analysis

The statistical package SPSS version 26.0 (SPSS Inc. Chicago, IL, US) was used. Demographic characteristics of the participants were analyzed using appropriate summary statistics for continuous and categorical variables. Further we used Welch’s *t*-test and Fisher’s Exact test to examine characteristics differences between EP-born and term-born controls. Results are reported with counts, proportions, means and standard deviations (SDs). The studies of HRQoL were parts of a comprehensive longitudinal assessment where statistical power had been calculated based on lung function data.

Sub-group analyses were preformed of the EP-born participants according to presence or absence of severe disability (healthy versus severe disability) defined by disabling cerebral palsy (CP), deafness or severe hearing loss, blindness or severe vision impairment.

We also preformed sub-group analyses of the EP-born participants by presence or absence of a neonatal history of bronchopulmonary dysplasia (BPD) which was defined by requirement for oxygen therapy at 36 weeks gestational age.

Mixed linear models were used to compare the EP and term-born groups. The matched structure of the EP and term-born participants (gender and age) and the repeated responses (17, 24 and 34 years for the HBSC-SCL and 24 and 34 years for the SF-36) were accounted for by assuming a covariance structure of unstructured correlation type. This method allows for contribution also from pairs with declines or who could not participate for some reason. The mean scores of the different domains were entered separately as dependent variables, whereas age and group were entered as independent variables with an interaction term to assess if group differences varied by age. Analyses were performed unadjusted and adjusted for gender, education and employment status. The criterion for statistical significance was p-value ≤ 0.05.

### Ethics

The study protocol was approved by the Regional Committee for Medical Research Ethics for Western Norway (Protocol no. 2017/628), and was performed in accordance with the Helsinki Declaration. All participants gave informed written consent for the assessments in adulthood.

## Results

**Fig. 1:**
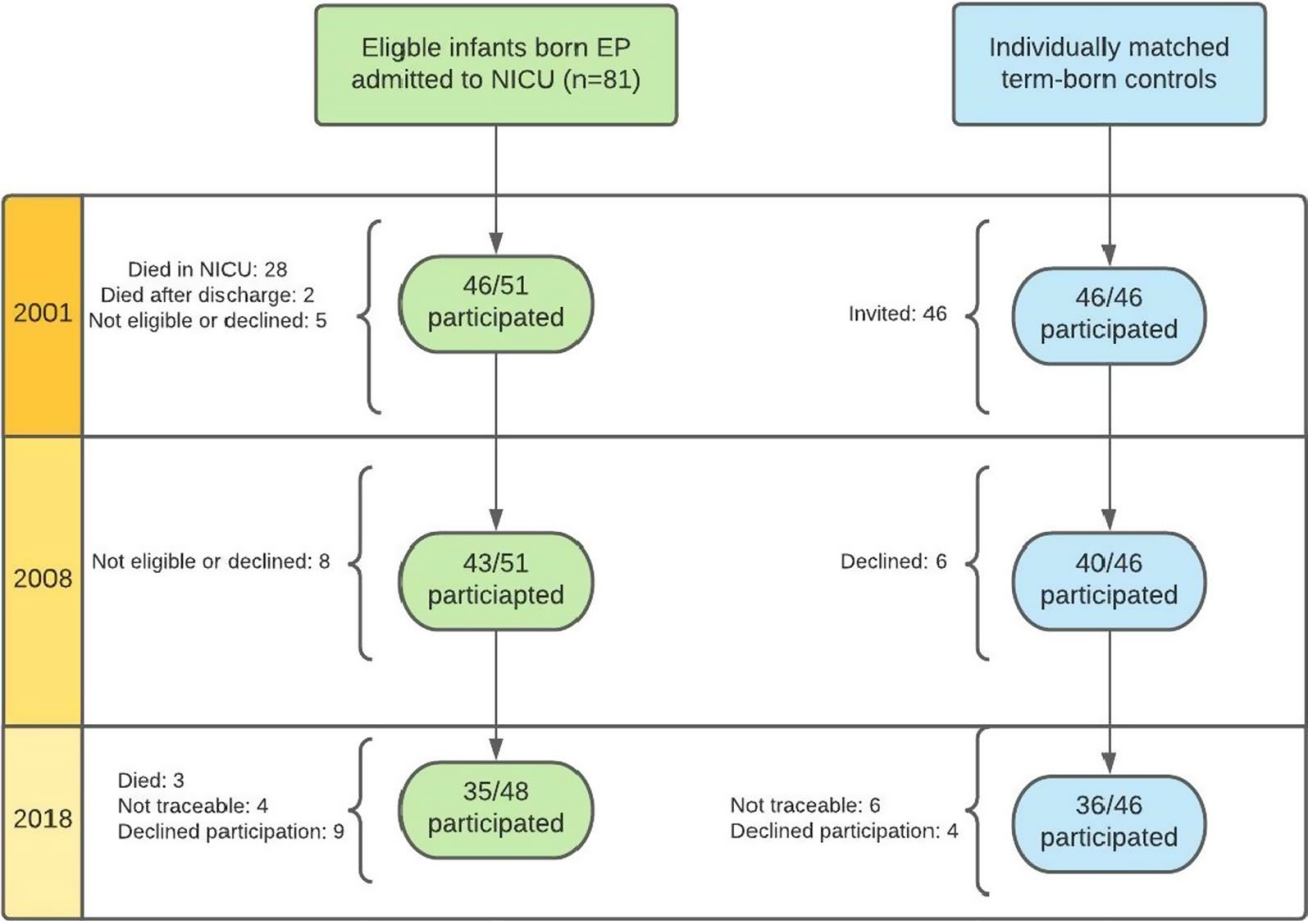
Flow chart of the 81 extremely preterm (EP) born subjects and 46 matched term-born controls at the three follow-up time points. *NICU* neonatal intensive care unit

Eighty-one EP-born were admitted to the NICU during the inclusion period, 51 (63%) were alive at the first follow-up in 2001–02, of whom 46 (90%) participated. At second (2008–09) and third (2018–20) follow-up, respectively 43/51 (84%) and 35/48 (73%) participated (Fig. 1). Corresponding numbers for the term-born controls were 46/46 (100%), 40/46 (87%) and 36/46 (78%), respectively.

### The first and second follow-up

Results from the first and second follow-up has been described in detail elsewhere [24].

### Demographic and clinical characteristics at 34 years of age (third follow-up)

Table 1 describes the participants’ birth characteristics, education, employment and civil status. The age range was 32–36 years. EP-born participants had lower educational level compared to term-born controls, seven EP-born had no daily work compared to one term-born.

**Table 1:** Characteristics of 45 extremely preterm subjects, with or without disability, and 46 term-born controls who were born in 1982–1985 in western Norway^a^

	EP-born severe disability	EP-born healthy	Term-born controls	p-value^b^	p-value^c^
Birth characteristics	n = 8	n = 37	n = 46	–	–
Male, *n (%)*	5 (62.5)	22 (55.0)	26 (56.5)	1.000	0.826
Gestational age, weeks, *mean (SD)*	26.9 (2.0)	27.5 (1.7)	–	–	–
Birth weight, grams, *mean (SD)*	917 (164)	1017 (194)	3441 (311)	< 0.001	< 0.001
Age first follow-up, year, *mean (SD)*	17.1 (1.6)	17.1 (1.1)	17.4 (1.3)	0.711	0.318
Age 2nd follow-up, year, *mean (SD)*	24.3 (1.7)	24.2 (1.2)	24.6 (1.3)	0.546	0.111
Age 3rd follow-up, year, *mean (SD)*	34.7 (1.6)	34.2 (1.3)	34.4 (1.3)	0.706	0.656
BPD moderate/severe, *n (%)*	2 (15.4)	11 (84.6)	0	–	–
*Neurosensory impairments, n (%)*					
Disabling CP, *n (%)*	4 (50.0)	0	0	–	–
Non-disabling CP, *n (%)*	2 (25.0)	4 (10.3)	0	–	–
Deaf, *n (%)*	2 (25.0)	0	0	–	–
Blind, *n (%)*	2 (25.0)	0	0	–	–
Characteristics at 34 years follow-up	n = 6	n = 28	n = 36		
* Highest educational attainment, n (%)*				0.395	0.436
College/University, ≤ 4 years	5 (83.3)	20 (71.4)	22 (61.1)		
College/University, > 4 years	1 (16.7)	8 (28.6)	14 (38.9)		
* Employment, n (%)*				0.007	0.159
Working or still in education	3 (50.0)	24 (85.7)	35 (97.2)		
Unemployed or disability pension	3 (50.0)	4 (14.3)	1 (2.8)		
* Martial status 34 years, n (%)*				< 0.001	0.112
Single	6 (100)	8 (28.6)	4 (11.4)		
Married/cohabitant	0	20 (71.4)	31 (88.6)		
* Having children, n (%)*	0	17 (61.0)	25 (69.0)	0.002	0.002

*BPD* bronchopulmonary dysplasia *CP* cerebral paresis *EP* extremely preterm *SD* standard deviation;

^a^Information were obtained from a general questionnaire and medical chart

^b^EP-born severe disability vs. term-born controls

^c^EP-born healthy vs. term-born controls

Differences were tested using Welch’s t-test for continuous variables and Fisher’s Exact Test for categorical variables

### HRQoL at 34 years of age (third follow-up)

Results are reported in Table 2. Due to missing data in pairs, the descriptive sub-group analyses were based on 5 pairs of EP-born with severe disability and 19 pairs of healthy EP-born with their respective term-born controls. The EP-born with severe disability scored clinical significant poorer than term-born in all domains, this was statistical significant for mental health. For the healthy EP-born participants, there were statistical significant differences vs. the term-born in several domains; vitality, role emotional and mental health. No statistical differences was found between EP-born with and without BPD (data not shown).

**Table 2:** Self-reported functional health and well-being, and Subjective health complaints at 34-years of age in 35 subjects born extremely preterm, with or without severe disability, and 35 matched term-born controls using the SF-36 questionnaire^a^ and HBSC-SCL^c^

	EP-born severe disability (n = 6)		EP-born healthy (n = 29)		Term-born controls (n = 35)		EP-born severe disability vs Term-born controls (n = 5)^b^	EP-born healthy vs Term-born controls (n = 19)^b^
	Mean (SD)	Range	Mean (SD)	Range	Mean (SD)	Range	Mean difference with 95% CI	Mean difference with 95% CI
*SF-36 domains*								
Physical Functioning	55.0 (41.1)	5–100	92.8 (9.6)	65–100	94.4 (11.0)	50–100	-55.0 (-112.0, 2.0)	-2.4 (-9.4, 4.7)
Role Physical	66.7 (51.7)	0–100	87.9 (29.6)	0–100	85.7 (32.8)	0–100	-40.0 (-108.0, 28.0)	1.3 (-19.0, 21.6)
Bodily Pain	63.0 (35.2)	22–100	74.8 (26.0)	22–100	78.5 (22.9)	22–100	-30.4 (-72.5, 11.7)	-9.9 (-26.7, 6.9)
General Health	51.6 (28.3)	25–87	69.9 (21.7)	20–100	75.7 (18.7)	25–100	-25.5 (-56.4, 5.4)	-11.1 (-24.1, 1.9)
Vitality	31.9 (20.6)	5–55	50.5 (21.7)	0–85	60.3 (19.1)	5–85	-15.7 (-20.4, 10.9)	**-19.2 (-35.0, -3.3)**
Social Functioning	72.9 (21.5)	50–100	81.5 (30.2)	0–100	89.6 (22.6)	0–100	-20.0 (-50.2, 10.2)	-17.1 (-37.1, 2.9)
Role Emotional	50.0 (54.8)	0–100	75.9 (38.7)	0–100	91.4 (26.0)	0–100	-40.0 (-108.0, 28.0)	**-22.8 (-45.6, -0.1)**
Mental Health	57.3 (24.9)	16–84	76.0 (17.2)	16–96	79.9 (16.7)	16–92	**-17.6 (-35.1, -0.2)**	**-10.4 (-20.9, 0.0)**
*HBSC-SCL variables*^*c*^								
HBSC total (0–32)	8.0 (8.2)	0–21	7.5 (7.8)	0–32	5.3 (6.1)	0–25	-0.3 (-5.9, 5.5)	**5.6 (0.6, 10.7)**
Somatic complaints, sub-score (0–16)	2.6 (2.9)	0–7	3.3 (4.1)	0–16	2.5 (2.8)	0–9	0.3 (-3.9, 4.4)	2.2 (-0.4, 4.8)
Psychological complaints, sub-score (0–16)	5.4 (5.6)	0–14	4.2 (4.6)	0–16	2.6 (3.7)	0–16	-0.5 (-2.6, 1.6)	**3.4 (0.6, 6.3)**

*CI* confidence interval *EP* extremely preterm *HBSC* Health Behaviour in School-aged Children-Symptom Checklist *SD* standard deviation

Bold results: p ≤ 0.05

^a^SF-36 (Short Form Health Survey-36), with possible domain scores from 0 to 100, higher score indicates better functional health and well-being

^b^The lower number is due to missing in pairs

^c^Higher score indicates more symptoms

### Subjective health complaints at 34 years of age (third follow-up)

Results are reported in Table 2. Due to missing data in pairs, the descriptive sub-group analyses were based on 4 pairs of EP-born with severe disability and 19 pairs of healthy EP-born with their respective term-born controls. There were no statistical significant differences between the EP-born subjects with severe disability and term-born controls in self-rated somatic and psychological health complaints. For healthy EP-born vs. term-born subjects, there were a significant difference in total subjective health complaints and the sub-score psychological complaints.

### Developmental of HRQoL from 24 to 34 years of age

**Fig. 2:**
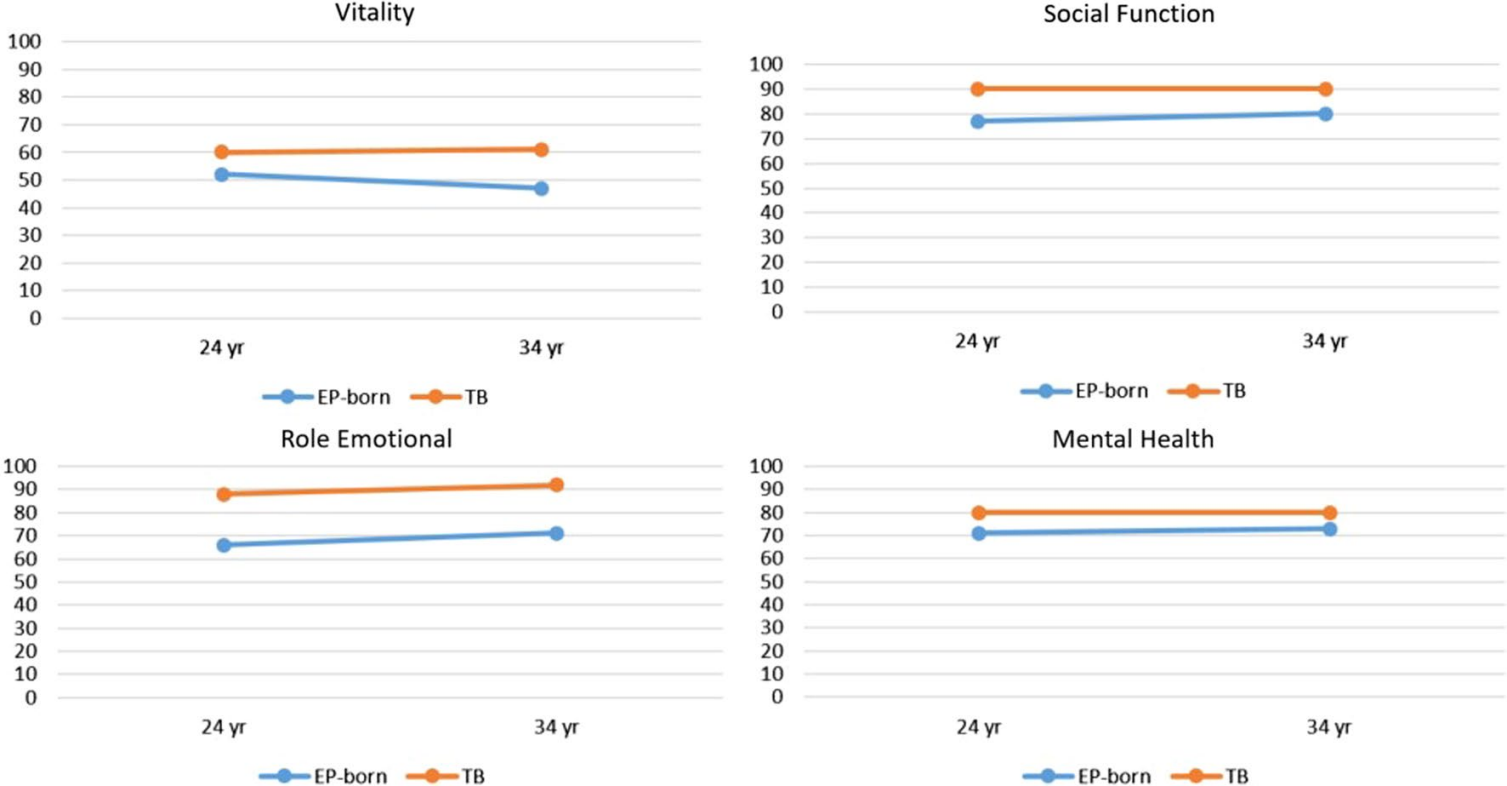
Group means of four self-reported HRQoL domains from 24 to 34 years of age in the extremely preterm (EP) born and the term-born (TB) participants according to SF-36. Higher score indicates better HRQoL, possible score from 0 to 100

Results from the mixed linear regression model are reported in Table 3. There were no significant interactions with age by group in the unadjusted and adjusted mixed effects analyses; i.e. development over the complete age span did not differ between the EP and term-born group. Thus, the results from the analyses without the interaction term were reported. Figure 2 illustrates the similar reporting of four domains at 24 and 34 years for EP and term-born participants.

**Table 3:** Pooled data of self-reported functional health and well-being at 24 and 34 year of age in subjects born extremely preterm and age- and gender matched term-born controls using the SF-36 questionnaire^a^

	Unadjusted^b^ Mean difference	Adjusted^c^ Mean difference
Response variable SF-36	EP-born verus Term-born estimate with 95% CI	EP-born versus Term-born estimate with 95% CI
Physical Functioning	− 8.93 ( − 14.56, − 3.30)**	− 1.60 ( − 7.18, 3.98)
Role Physical	− 5.54 ( − 15.11, 4.02)	− 0.98 ( − 11.00, 9.04)
Bodily Pain	− 8.80 ( − 16.48, − 1.13)**	− 2.81 ( − 11.13, 5.50)
General Health	− 7.23 ( − 13.08, − 1.39)**	− 5.94 ( − 13.19, 1.31)
Vitality	− 11.15 ( − 16.84, − 5.47)***	− 7.34 ( − 14.08, − 0,61)*
Social Functioning	− 11.97 ( − 19.55, − 4.40)**	− 10.11 ( − 18.10, − 2.12)*
Role Emotional	− 22.11 ( − 33.19, 11.04)***	− 15.48 ( − 27.40, − 3.55)**
Mental Health	− 8.83 ( − 13.86, − 3.79)***	− 6.12 ( − 11.87, − 0.36)*

*CI* confidence interval *EP* extremely preterm *SD* standard deviation

^*^p ≤ 0.05; **p ≤ 0.01; ***p ≤ 0.001

^a^SF-36: Short Form Health Survey-36, with possible domain scores from 0 to 100, higher score indicates better functional health and well-being

^b^Mixed effects linear models including EP-born and term-born and age group. No significant difference between age 24 and 34 was found and therefore the results without age in the model are reported to increase the statistical power

^c^Mixed effects linear models adjusted for gender, education level, employment status and age were used in the model to assess differences in the SF-36 scores between 24 and 34 years, and between the groups at each age, including a group by age interaction

^d^College/University under or over 4 years

^e^Not working/retirement income or working

In the unadjusted mixed effects analyses, there were statistical significant differences between the EP-born and term-born participants over the ten years age span in seven of the eight domains (physical functioning, bodily pain, general health, vitality, social functioning, role emotional, and mental health). Adjusted for gender, education and employment status, the group differences remained, with EP-born scoring statistical significantly lower in vitality, social functioning, role emotional, and mental health.

### Developmental of subjective health complaints from 17 to 34 years of age

**Fig. 3:**
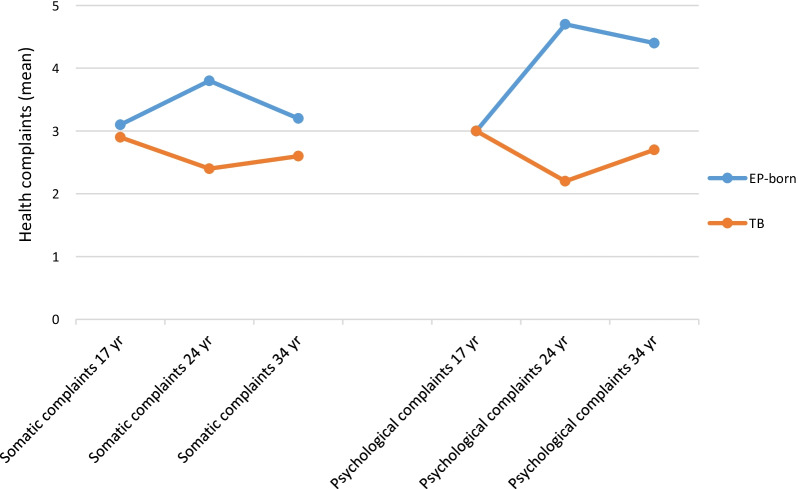
Group means of self-reported somatic and psychological health complaints from 17 to 34 years of age in the extremely preterm (EP) born and the term-born (TB) participants according to the Health Behaviour in School-aged Children–Symptom Checklist (HBSC-SCL). Higher score indicates more complaints, possible sub score from 0 to 16 on sub scores

Results from the mixed linear regression model are reported in Table 4. For somatic complaints, the adjusted mean estimated difference between EP-born and term-born participants was 0.76, but not significant, and this difference remained stable over the age groups (test for interaction p = 0.321), although it was numerically lowest at 17 years and highest at 24 years. The same pattern was shown for psychological complaints, but in this case the variation between the ages was statistical significant (interaction p = 0.027) with adjusted mean estimated differences of 0.00, 2.53 and 1.86 at 17, 24 and 34 years, respectively. The patterns for the two sub-scores are depicted in Fig. 3. The total score followed the same pattern, but the interaction was not significant (p = 0.058).

**Table 4:** Differences in subjective health complaints at 17, 24 and 34 years between subjects born extremely preterm, and matched^a^ controls born at term provided by the Health Behaviour School-aged Children-Symptom Checklist (HBSC-SCL)^b^ and estimated by mixed linear models^c^

	Unadjusted	Adjusted				
Response variable HBSC-SCL	Mean difference EP-born versus Term-born Estimate 95% CI	At age 17 years Mean difference EP-born versus Term-born Estimate 95% CI	At age 24 years Mean difference EP-born versus Term-born Estimate 95% CI	At age 34 years Mean difference EP-born versus Term-born Estimate 95% CI	Group by age p-value	Mean difference EP-born versus Term-born Estimate 95% CI^d^
Total score	2.21 (0.84, 3.59)**	0.22 ( − 1.97, 2.40)	4.01 (1.68, 6.34)	2.85 (0.26, 5.44)	0.058	2.36 (0.62, 4.11)
Somatic complaints sub-score	0.85 (0.14, 1.55)**	0.22 ( − 0.91, 1.34)	1.47 (0.27, 2.67)	0.97 ( − 0.37, 2.31)	0.321	0.76 ( − 0.15, 1.67)
Psychological complaints sub-score	1.37 (0.54, 2.19)***	0.00 ( − 1.31, 1.31)	2.53 (1.14, 3.92)	1.86 (0.32, 3.40)	0.027*	1.63 (0.61, 2.65)

*CI* confidence interval *EP* extremely preterm *HBSC-SCL* Health Behaviour in School-aged Children-Symptom Checklist *SD* standard deviation

*p ≤ 0.05; **p ≤ 0.01; ***p ≤ 0.001

^a^Matched for gender and age

^b^With possible score from 0 to 32 on total HBSC and 0–16 on sub-scores. Higher score indicates more symptoms

^c^Mixed linear models were used to assess differences in the HBSC-SCL scores between the groups at each age 17, 24 and 34 years, by including a group by age interaction ^d^Adjusted for gender, education level, employment status and age in models without group by age interaction

## Discussion

In this population-based longitudinal study, HRQoL and subjective health complaints were repeatedly assessed from 17 to 34 years of age in EP-born adults and compared with a matched term-born control group. HRQoL was poorer in EP-born without severe disabilities at 34 years, and their scores were unchanged from previous assessments at 24 years of age. EP-born also scored poorer regarding subjective health complaints at 34 years, but development from 24 years was positive (less complaints) for sub-scores addressing psychological issues. EP-born with severe disabilities were few, which prevented firm conclusions for this group.

HRQoL in the EP-born participants of this study was poorer at 34 years of age compared to matched term-born controls, and had remained unchanged over the age span covering the preceding decade from 24 to 34 years of age. The scores of the control group were in line with observations made in a large study covering a comparable background population, as was also their stable development during this same age span [31]. The scores for our EP-born were in agreement with the few longitudinal studies that have been carried out in preterm-born adults. Saigal et al. [23] found the same poor HRQoL scores over time, measured by the Health Utilities Index Mark 3 (HUI3), as did also a Dutch study which also applied the HUI3 during the period 19 to 28 years [22]. Our results are also in line with some cross-sectional studies from early adulthood [12, 34], although there are inconsistencies in the literature with some studies reporting similar HRQoL in preterm born and term-born peers [21, 35–37].

Causal relationships between EP birth and poor HRQoL cannot easily be extracted from this dataset, and we are left with speculations. In their mid-twenties, most young adults find themselves in a process of establishing an independent life, about to finish education, and/or in search of a job or future career. Later, in their mid-thirties, most will be in more permanent work and social arrangements, perhaps with children on their own. We found that by the age of 35 years, fewer EP-born than term-controls had established relationships with another person, their level of education was lower, and more were unemployed or received some kind of financial support. As such, they seemingly had achieved a different level of independent life than their term-born peers and these and all these sociodemographic factors are known to be related to poorer HRQoL [38–41]. However, the association between these variables are unknown, and poor HRQoL might represent both a cause and a consequence of a disadvantageous life situation. For example, some authors argue that level of education should be considered an outcome after preterm birth [42], whereas others disagree [43]. In our dataset, group differences in the mental health domains remained in analyses adjusted for educational level and employment status, indicating that these deficits could not be explained by these factors alone.

EP-born with severe disabilities were rare in our cohort, which prevented firm conclusions for this group; however, their scores were numerically poorer for all HRQoL domains when compared with the control group as well as the EP-born without severe disabilities. For healthy EP-born, poor HRQoL scores were particularly evident on the domains addressing vitality, role emotion and mental health. These domains reflect experiences of depression, difficulties at work or in social contexts due to emotional problems, and feelings of fatigue. Decreases versus the control group were not only of statistical significance; they also exceeded levels regarded clinically significant [29], and therefore likely to be of major personal importance for those affected. As HRQoL represent a fundamental measure of health [18] these findings are also of obvious societal interest, given the high number of EP-born currently entering adulthood [44]. Thus, it is vitally important to understand what may contribute to this situation.

Contrasting HRQoL, development from 24 to 34 years regarding health complaints was positive (less complaints) in our EP-born group. One way of interpreting the development from 24 to 34 years is by a shift in internal standards and how questions are valued; a mechanism referred to as response shift [45]. This would imply that EP-born well into adulthood live better with complaints they had also as young adults at 24 years. If that is correct, this mechanism has come into play only for psychological complaints, not for somatic complaints and also not for HRQoL. We argue that this is a phenomenon that should be considered and investigated also in EP-born populations, as it has been studied in patients with severe chronic conditions and cancer patients [46].

The major strengths of this study were the longitudinal design that takes us well into adulthood, participation of the same matched term-born control group throughout the complete study period, a relatively high participation rate, and the use of standardised and validated questionnaires. Recruitment of the term-born control group was based on the ‘next born subject principle’, minimising the risk of selection bias. There were rather wide confidence intervals on the different HRQoL domains, irrespective of disabilities or not, suggesting that our EP-born participants were rather heterogeneous. This may challenge the generalisability, but may also be viewed as reflections of heterogeneous outcomes after EP birth and thus traits to be expected in studies like the present. A relatively small overall sample size decreased statistical power, challenged inclusion of possible confounding variables, and increased the risk of type-two errors, particularly relevant to the comparisons between EP-born with and without severe disability. The study was part of an extensive follow-up investigating a range of variables after EP birth, and power calculations had been done 20 years ago focusing on lung function. Post hoc power calculations were not carried out [47], and instead we provide 95% confidence intervals and *p*-values to quantify the uncertainty. Another limitation is that RAND-36 was used at the second follow-up, compared to SF-36 at the first. As mentioned in the Methods section, RAND-36 is considered equivalent to the SF-36, and we do not believe this had an impact on our results, but important to have in mind.

Our participants were born in the early 1980s and represent a “pioneer generation” in the sense that most infants born at this early stage during preceding decades had died. Survival rates have increased since the 1980s, particularly in the low gestational age range, and increasingly more survivors of extreme immaturity are currently approaching adulthood. Thus, we need sufficiently powered follow-up studies to explore potential cohort effects, and we should conduct in-depth qualitative studies to investigate the lived experience of EP-born individuals. The longitudinal data provided in this present study suggest there may be a window of opportunity during their late teens with advantageous self-reported scores for quality of life and health complaints that perhaps can be utilized for interventions.

## Conclusion

The study demonstrates that 34-years-old EP-born adults had poorer HRQoL than term-born peers, especially in the mental health domains, and that these deficits had remained unchanged during the preceding decade. Thus, one may fear that this situation may endure even further with unknown consequences. Similarly, healthy 34-year-old EP-born with no major disabilities had more subjective health complaints compared to term-born peers; however, development during the preceding decade had improved for the psychological subscale, suggesting either real improvement or some sort of shift in internal standards on how questions are valued.

## Data Availability

In accordance with the approvals granted for this study by The Regional Committee on Medical Research Ethics, the data files are stored securely and in accordance with the Norwegian Law of Privacy Protection. The data file cannot be made publicly available as this might compromise the respondents’ privacy. A subset of the data file with anonymized data can be made available to interested researchers upon reasonable request to Thomas Halvorsen (thomas.halvorsen@helse-bergen.no), providing Norwegian privacy legislation and GDPR are respected, and that permission is granted from The Norwegian Data Inspectorate and the data protection officer at Haukeland University Hospital.

## Abbreviations

EP: Extremely preterm
NICU: Neonatal intensive care unit
GA: Gestational age
BW: Low birthweight
HRQoL: Health-related quality of life
SF-36: Short-Form Health Survey
HBSC-SCL: Health behaviour in school-aged children-symptom check list
SD: Standard deviation
CP: Cerebral palsy
BPD: Bronchopulmonary dysplasia
HUI3: Health utilities index mark 3
